# Transcriptome Profiling Reveals Interplay of Multifaceted Stress Response in *Escherichia coli* on Exposure to Glutathione and Ciprofloxacin

**DOI:** 10.1128/mSystems.00001-18

**Published:** 2018-02-13

**Authors:** Manish Goswami, Akkipeddi Venkat Satya Surya Narayana Rao

**Affiliations:** a Molecular Biology Division, Bhabha Atomic Research Centre, Trombay, Mumbai, India; b Homi Bhabha National Institute, Anushaktinagar, Mumbai, India; University of California, San Diego

**Keywords:** DNA damage, acid resistance, antibiotic resistance, cellular redox status, redox function, stress response, transporters, virulence

## Abstract

We have previously reported that supplementation of exogenous glutathione (GSH) promotes ciprofloxacin resistance in *Escherichia coli* by neutralizing antibiotic-induced oxidative stress and by enhancing the efflux of antibiotic. In the present study, we used a whole-genome microarray as a tool to analyze the system-level transcriptomic changes of *E. coli* on exposure to GSH and/or ciprofloxacin. The microarray data revealed that GSH supplementation affects redox function, transport, acid shock, and virulence genes of *E. coli*. The data further highlighted the interplay of multiple underlying stress response pathways (including those associated with the genes mentioned above and DNA damage repair genes) at the core of GSH, offsetting the effect of ciprofloxacin in *E. coli*. The results of a large-scale validation of the transcriptomic data using reverse transcription-quantitative PCR (RT-qPCR) analysis for 40 different genes were mostly in agreement with the microarray results. The altered growth profiles of 12 different *E. coli* strains carrying deletions in the specific genes mentioned above with GSH and/or ciprofloxacin supplementation implicate these genes in the GSH-mediated phenotype not only at the molecular level but also at the functional level. We further associated GSH supplementation with increased acid shock survival of *E. coli* on the basis of our transcriptomic data. Taking the data together, it can be concluded that GSH supplementation influences the expression of genes of multiple stress response pathways apart from its effect(s) at the physiological level to counter the action of ciprofloxacin in *E. coli*.

**IMPORTANCE** The emergence and spread of multidrug-resistant bacterial strains have serious medical and clinical consequences. In addition, the rate of discovery of new therapeutic antibiotics has been inadequate in last few decades. Fluoroquinolone antibiotics such as ciprofloxacin represent a precious therapeutic resource in the fight against bacterial pathogens. However, these antibiotics have been gradually losing their appeal due to the emergence and buildup of resistance to them. In this report, we shed light on the genome-level expression changes in bacteria with respect to glutathione (GSH) exposure which act as a trigger for fluoroquinolone antibiotic resistance. The knowledge about different bacterial stress response pathways under conditions of exposure to the conditions described above and potential points of cross talk between them could help us in understanding and formulating the conditions under which buildup and spread of antibiotic resistance could be minimized. Our findings are also relevant because GSH-induced genome-level expression changes have not been reported previously for *E. coli*.

## Abstract (precis)

The emergence and spread of multidrug-resistant bacterial strains have serious medical and clinical consequences. In addition, the rate of discovery of new therapeutic antibiotics has been inadequate in last few decades. Fluoroquinolone antibiotics such as ciprofloxacin represent a precious therapeutic resource in the fight against bacterial pathogens. However, these antibiotics have been gradually losing their appeal due to the emergence and buildup of resistance to them. In this report, we shed light on the genome-level expression changes in bacteria with respect to glutathione (GSH) exposure which act as a trigger for fluoroquinolone antibiotic resistance. The knowledge about different bacterial stress response pathways under conditions of exposure to the conditions described above and potential points of cross talk between them could help us in understanding and formulating the conditions under which buildup and spread of antibiotic resistance could be minimized. Our findings are also relevant because GSH-induced genome-level expression changes have not been reported previously for *E. coli*.

## INTRODUCTION

Antibiotics are weapons of choice in the fight against infectious bacterial diseases to eliminate the causative pathogens or to slow their growth such that host defense mechanisms can clear the infection from the host (1). Antibiotic exposure triggers a bacterial adaptive response to offset the consequences of the exposure to the given antibiotic. The adaptive response generated to overcome the given stress in a bacterial cell for its better survival and increased fitness is termed the bacterial stress response (2). Bacteria respond to such changes by altering their gene expression pattern, which helps in adjusting the cellular physiology and metabolism to accommodate the new condition(s), thereby protecting the bacteria against cell damage or death (2). Different groups of antibiotics target diverse and seemingly unrelated subcellular bacterial targets, thereby generating an unambiguous adaptive stress response (3) with a signature of the specific antibiotic class; e.g., exposure of *Escherichia coli* cells to fluoroquinolone, aminoglycoside, and β-lactam leads to the generation of SOS (4), heat shock (5), and cell envelope (6) stress responses, respectively.

In light of the antibiotic discovery pipeline running dry along with the looming threat of the advent of a postantibiotic era due to rapid emergence and spread of multidrug-resistant (MDR) pathogens (7), it is incumbent upon us to safeguard the utility of the currently available chemotherapeutic antibiotics. Consequently, knowledge about molecular mechanisms of antibiotic action, related bacterial responses, factors modulating their activity, and antibiotic resistance could help us in understanding the conditions where resistance is selected and persists. This knowledge could be useful for development of improved antibacterial substances and therapeutic regimens to help us in keeping pace with the remarkable adaptability of pathogenic bacteria.

**FIG 1:**
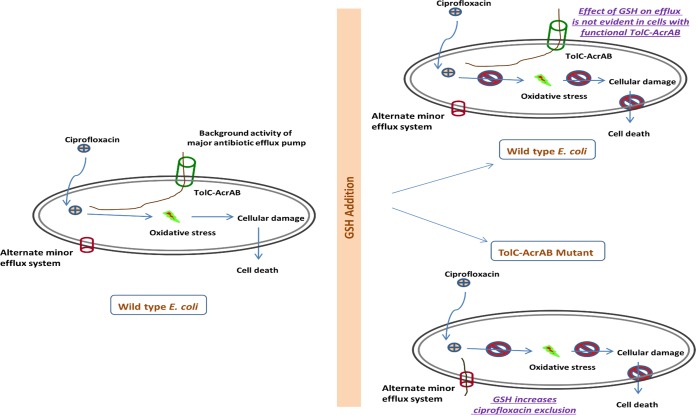
A schematic diagram showing the effect of glutathione supplementation and the role of previously reported metabolic pathways to counter the antibacterial effect of ciprofloxacin in wild-type and TolC-AcrAB mutant strains of *Escherichia coli*.

We have previously shown that supplementation of exogenous glutathione (GSH) in *E. coli* reverses the effect of ciprofloxacin by neutralizing the oxidative stress involved in its antibacterial action (8). We further established recently that GSH supplementation promotes ciprofloxacin resistance by increasing its efflux from *E. coli* (9). Therefore, GSH-mediated abrogation of ciprofloxacin-induced bacterial killing can be attributed to both (i) decreased oxidative stress and (ii) increased antibiotic efflux from *E. coli* as shown in Fig. 1. Since GSH was found to influence more than one biological process here, we were curious to understand the effect of GSH supplementation at the system level as well as in context with the phenotype mentioned above for *E. coli*. Consequently, genome-level *E. coli* expression changes in response to GSH supplementation, subinhibitory ciprofloxacin exposure, and GSH-mediated abrogation of bacterial killing by ciprofloxacin were analyzed. Notably, genome-wide expression changes in antibiotic-resistant *E. coli* have not been adequately explored, with the exception of a previous study using a clinical isolate (10). Moreover, the genome-level expression changes in bacteria with respect to different types of triggers leading to antibiotic resistance are still unknown. In the present study, we conducted genomic expression profiling of *E. coli* MG1655 with GSH and/or ciprofloxacin using DNA microarrays. The data highlight an interplay of multiple underlying stress response pathways under conditions of exposure of *E. coli* cells to GSH and/or ciprofloxacin. The DNA microarray results were further validated for all the genes (*n* = 40) showing a ≥5.0-fold change in expression using reverse transcription-quantitative PCR (RT-qPCR). In addition, we determined the functional significance of all the above-mentioned genes with respect to the GSH-mediated phenotype by monitoring the effect of different *E. coli* gene deletion mutants on their growth profiles in the presence of GSH and/or ciprofloxacin. Since our transcriptomic data suggested that GSH supplementation promotes the expression of acid shock genes, we also analyzed the effect of exogenous GSH on acid stress adaptation of *E. coli* cells in the present study.

## RESULTS

### Genome-scale transcriptomic data for *E. coli* MG1655 in response to exogenous GSH and/or ciprofloxacin.

**FIG 2:**
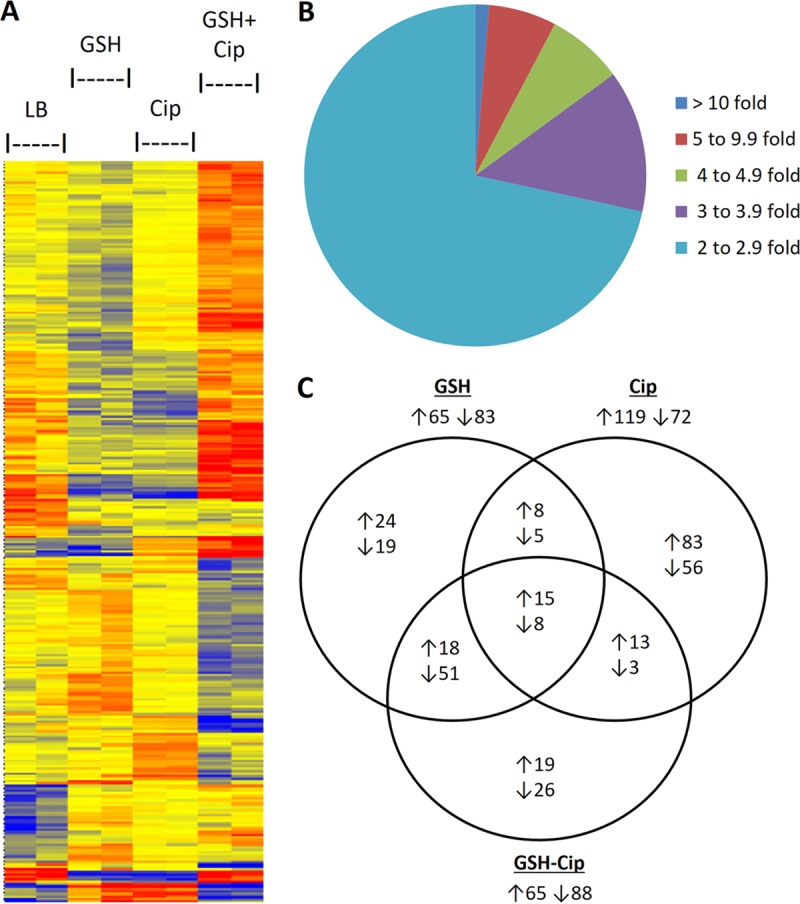
(A) Heat map of microarray gene expression. Comparisons of gene expression patterns of the whole transcriptome of MG1655 under 4 different exposure conditions are shown. The gradient is representative of the expression differences between the samples, where red indicates upregulated expression of the given gene and blue indicates downregulated expression. Exposure conditions are shown at the top of the figure. (B) Pie chart showing the relationship between the fold change value and the number of genes for 609 genes showing a ≥2.0-fold change in microarray analysis. (C) Venn diagram of the number of genes with ≥2.0-fold differential expression during GSH and/or ciprofloxacin exposure at concentrations of 10 mM GSH, 3 ng/ml (subinhibitory concentration) of ciprofloxacin, and 10 mM GSH with 50 ng of ciprofloxacin/ml (inhibitory concentration). Numbers of upregulated and downregulated genes are indicated next to ↑ and ↓ arrows, respectively.

We first examined the genome-level expression changes in *E. coli* strains subjected to sub-MIC ciprofloxacin exposure, GSH supplementation, and GSH-mediated abrogation of bacterial killing caused by ciprofloxacin. Accordingly, 4 different groups of growing MG1655 cells, including (i) a control group, (ii) a group exposed to 10 mM GSH, (iii) a group exposed to 3 ng/ml ciprofloxacin, and (iii) a group exposed to 10 mM GSH and 50 ng/ml ciprofloxacin, were subjected to microarray analysis as described in Materials and Methods. The details of the microarray data can be retrieved from the NCBI Gene Expression Omnibus (GEO) database (GSE93896). A heat map of the microarray expression data is shown in Fig. 2A. Data analysis was based on 5 different types of comparisons, including (i) control versus GSH, (ii) control versus ciprofloxacin (iii), control versus GSH plus ciprofloxacin, (iv) ciprofloxacin versus GSH plus ciprofloxacin, and (v) GSH versus GSH plus ciprofloxacin. To start with, the total number of probe sets detected for the experiment was 10,208, which became 7,603 after the data preprocessing and normalization steps. A total of 1,760 gene/probe sets among the 7,603 were found to be differentially expressed (significant at *P* = ≤0.05). Finally, 609 genes were found to exhibit a ≥2.0-fold change in one of the comparisons defined above (see Table S1 at http://www.barc.gov.in/publications/mSystems00001-18/). An inverse relationship between the fold change value and the number of genes affected was evident from the pie chart shown in Fig. 2B. The similarities and differences in the gene expression patterns seen under 3 different exposure conditions have been depicted in the form of a Venn diagram in Fig. 2C for all the genes showing a ≥2.0-fold change in expression.

Hierarchical clustering by function of gene product performed using gene ontology analysis of differentially expressed genes (DEG) revealed that biological, cellular, and molecular functions of *E. coli* are affected by the exposure conditions mentioned above. The group of genes affected by GSH exposure comprised those corresponding to redox functions (e.g., *adhE*, *azoR*, *fecA*, *frdB*, and *yeiH*), acid shock (e.g., *asr*, *ogrK*, *ydeO*, and *yegR*), transport (e.g., *lamB emrK*, *srlB*, and *yebF*), and virulence functions (*emrK*, *nmpC*, *yebF*, and *ypdI*) (Table 1). Ciprofloxacin exposure led to increased expression of repair/recombination and cytokinesis genes (e.g., *lexA*, *recA*, *recN*, *dinB*, and *sulA*) (Table 1). On the other hand, the microarray gene expression pattern for the GSH-plus-ciprofloxacin group showed that the levels of expression were largely moderate (with the exception of a few genes) compared to the results from both the GSH and ciprofloxacin groups (Table 1). Among the five comparison groups mentioned above, expression of a total of 41 MG1655 genes was found to be induced or repressed ≥5.0-fold during microarray expression analysis (Table 1).

**TABLE 1:** Change in expression of 41 genes compared to LB (control condition) detected by microarray analysis^a^

Gene name	Avg fold change in expression compared to the control under given exposure condition			Cellular location of the affected gene per the Ecocyc database (47)
	GSH	Cip	GSH + Cip	
*adhE*	**4.3↓**	1.5↑	**5.9↓**	Cytosol, membrane
*asr*	9.5↑	**1.6↓**	7.2↑	Periplasmic location
*azoR*	**12.6↓**	**1.2↓**	**9.6↓**	Cytosol
*csrB*	**5.9↓**	1.8↑	**4.9↓**	Cytosol
*dinB*	1.01↑	5.7↑	2.3↑	Cytosol
*emrK*	3.8↑	**1.4↓**	3.5↑	Inner membrane
*fbaA*	**2.4↓**	2.09↑	**2.02↓**	Inner membrane
*ffs*	**1.9↓**	**6.6↓**	**1.5↓**	Cytosol
*fecA*	**4.9↓**	1.04↑	**3.7↓**	Outer membrane
*fimH*	1.4↑	**2.9↓**	1.7↑	Extracellular space, pilus
*frc*	**3↓**	1.4↑	**3.7↓**	Cytosol
*frdB*	**3↓**	1.5↑	**3.6↓**	Inner membrane, cytosol
*gcvP*	**3.4↓**	1.5↑	**3.06↓**	Cytosol
*glpC*	4.6↑	**1.3↓**	5.3↑	Cytosol, inner membrane
*lamB*	**3.1↓**	1.4↑	**5.4↓**	Outer membrane
*lexA*	**1.5↓**	4.4↑	2.3↑	Cytosol
*manX*	**1.9↓**	1.7↑	**3.3↓**	Inner membrane, cytosol
*manY*	**2.2↓**	1.6↑	**4.2↓**	Inner membrane
*manZ*	**2.8↓**	1.1↑	**5.6↓**	Inner membrane
*nmpC*	**3.2↓**	1.5↑	**6.8↓**	Inner membrane
*ogrK*	6.6↑	2.1↑	8.2↑	Cytosol
*oxc*	5.7↑	**1.1↓**	3.4↑	Not reported
*recA*	**1.4↓**	7.5↑	3.3↑	Cytosol
*recN*	**1.1↓**	11.8↑	3.6↑	Cytosol
*srlB*	**2.1↓**	1.6↑	**3.6↓**	Cytosol
*srlD*	**1.2↓**	3.2↑	**1.5↓**	Cytosol
*srlE*	**1.6↓**	3.9↑	**2.2↓**	Inner membrane
*srlR*	**1.01↓**	4.01**↑**	**1.3↓**	Cytosol
*sulA*	**1.3↓**	7.1↑	3.5↑	Inner membrane
*tdcD*	**1.5↓**	**2.1↓**	**2.4↓**	Cytosol
*tnaA*	**2.2↓**	2.1↑	**2.4↓**	Cytosol, membrane
*treC*	**4.2↓**	1.7↑	**3.6↓**	Cytosol
*ybiJ*	**5.8↓**	1.4↑	**5.1↓**	Periplasmic space
*ydeO*	5.3↑	**1.3↓**	**5.5↓**	Cytosol
*yebF*	1.02↑	5.2↑	4.1↑	Periplasmic space
*yegR*	11.9↑	**1.8↓**	11.7↑	Not reported
*yeiH*	4.4↑	1.08↑	6.8↑	Inner membrane
*yfdX*	9.8↑	**1.2↓**	10.9↑	Periplasmic space
*yhaK*	**5.8↓**	**1.03↓**	**7↓**	Cytosol
*yjiY*	**6.8↓**	**1.5↓**	**7.8↓**	Inner membrane
*ypdI*	5.5↑	1.5↑	5.9↑	Periplasmic space/ Inner membrane

^a^ Font in lightface with an upward arrow represents upregulation, and font in boldface with a downward arrow represents downregulation of the given gene product.

### Multigene validation of transcriptomic data using RT-qPCR.

**FIG 3:**
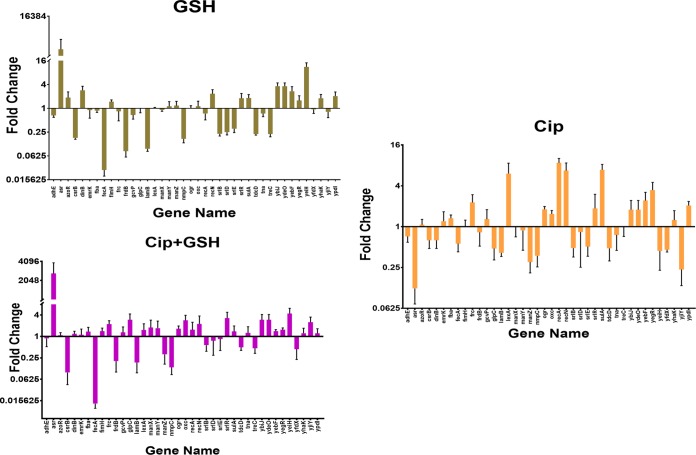
Changes in gene expression level of 40 genes of MG1655 on exposure to GSH and/or ciprofloxacin by RT-qPCR analysis. The 40 genes are the same as those which exhibited ≥5.0-fold-altered expression in microarray analysis. Mid-logarithmic-phase bacterial cells were used for RT-qPCR analysis per the details mentioned in Materials and Methods. Data represent expression levels of different genes in the presence of (A) 10 mM GSH, (B) 3 ng of ciprofloxacin/ml, and (C) 10 mM GSH with 30 ng of ciprofloxacin/ml. Each bar represents the ratio of the expression level of that gene in the presence of the given exposure condition to the expression level under the control conditions using *mdoG* as an external reference gene. The bar diagram shown here represents data from three different biological replicates (*n* = 3). Error bars represent ± standard errors of the means (SEM).

Except for the *ffs* gene (which was too small for optimum primer designing), all 41 of the genes showing ≥5.0-fold-altered expression in microarray analysis were selected for confirmation of the whole-genome transcriptomic data (see Table 2 for details) using the RT-qPCR approach. The RT-qPCR expression profile corroborated the microarray results, as the data from 15 of 40 genes fully supported the microarray data in terms of induction or repression pattern for all 3 exposure conditions. Among the remaining 25 genes, an initial ≥2.0-fold cutoff value for microarray data resulted in a perfect match with the qPCR data under all 3 exposure conditions for 13 more genes. Generally, the magnitudes of change in gene expression were found to be different upon comparison of the microarray data with the qPCR data, though the trends largely matched for the two techniques; e.g., the levels of GSH-mediated repression of *nmpC* and *lamB* were found to ~3.2-fold and ~3.1-fold using the microarray and ~6.25-fold and ~10.6-fold using qPCR. The biggest differences in the fold change values were noted for *asr*, whose expression was found to be induced by ~9.5-fold and ~7.2-fold by GSH and GSH plus ciprofloxacin, respectively, by microarray analysis and ~9,000-fold and ~2,600-fold by qPCR analysis. The *asr* repression values after ciprofloxacin exposure were ~1.6 and ~8.3 based on the microarray and qPCR data, respectively. Partial matches or no matches between microarray and qPCR data in terms of expression patterns were observed for remaining 12 genes (including *azoR*, *emrK*, *glpC*, *manY*, *manZ*, *ogrK*, *oxc*, *ybiJ*, *ydeO*, *yegR*, *yfdX*, and *yjiY*). Moreover, it was evident from the data presented in Fig. 3 that the RT-qPCR data for many of the genes showed similar expression profiles for GSH and GSH plus ciprofloxacin, which contrasted with the results seen with *E. coli* cells exposed to ciprofloxacin.

**TABLE 2:** List of *E. coli* MG1655 genes used for expression analysis using RT-qPCR^a^

Serial no.	Gene name(s)	Gene function	NCBI gene ID	5′ primer	3′ primer	Amplicon size (bp)
1	*acpD*/*azoR*	Thiol stress protection	947569	GCACTCCGCTGATGAAAT	GGTGCCGCAATAACGATAA	189
2	*adhE*	Alcohol dehydrogenation	945837	GGGTTCCCAGTTCCATATTC	GTCGGCAATTTCAGCATAAC	154
3	*asr*	Acid shock resistance	945103	TGCTATGGGTCTGTCTTCT	GGGCTTTCTGTTCAGCTT	188
4	*csrB*	Carbon storage regulation	2847719	CCAGGATGGAGAATGAGAAC	GCAGCATTCCAGCTACTT	190
5	*dinB*	Adaptive mutation induction	944922	CGCCTCCGACATGAATAA	GCATCACCAGATCACACTT	179
6	*emrK*	Multidrug efflux transportation	946840	TTAAACCGTCAGCCACAAG	CGGTACGACAGCCATTAAC	168
7	*fba*/*fbaA*	Glycolysis aldol condensation catalyzing	947415	GCCGGAAGACGTTGATTAC	CGGCAGGTTGTGTTTCTT	172
8	*fecA*	Ferric citrate uptake for porin formation	946427	CGTACAGTACAGCCAGATTG	GGTTGGAGTCGTACTGATTG	158
9	*fimH*	Receptor recognition and fimbrial adhesion	948847	CTTATGGCGGCGTGTTAT	GCTCACAGGCGTCAAATA	152
10	*frdB*	Iron-sulfur fumarate reduction	948666	GCGAAGTATCACCAGTTCTC	GCCATACGCTCCTTCTTAC	161
11	*gcvP*	Glycine decarboxylation	947394	GCGCAACAGCAAGAAATG	GTAACCCATGCCGATGTAAG	198
12	*glpC*	Anaerobic glycerol-3-phosphate dehydrogenation	946735	ACCAGGTCGCTTTCTTTC	CCTGTTTGCGTGCTTTATC	174
13	*lamB*	Maltose diffusion facilitation	948548	ATGCACGTTCCGGTATTG	AGCTCTTATCGCCCTCTT	156
14	*lexA*	Transcriptional repression of SOS regulon	948544	CTCATCCGTGATCACATCAG	GCAACCCTTCTTCCTCTTC	190
15	*manX*	2-Deoxyglucose phosphorylation	946334	CGTGCTGTTTCTCGTTGATA	TCACGGCCTGTTTCTACT	189
16	*manY*	Mannose PTS permease formation	946332	GTTTCACCGTCCGCTAAT	CTCTGATGACCTGCGATAAC	189
17	*manZ*	Mannose PTS permease formation	946342	CTGGTTGTCTCTCGCATTAC	CGATACCGATGACGAAGAAG	181
18	*mdoG*/*opgG*	Membrane-derived oligosaccharide synthesis	945005	GTGGATGTGCAGTCGAAA	CATTACCGGCATGGATAGAG	163
19	*nmpC*	Outer membrane porin formation	946786	ACTACGGCTCCATCGATTA	CCATCAACCAGACCAAAGAA	178
20	*ogrK*	Bacteriophage P2 late transcription control	945404	CAAGCCGCTATATCACTGAC	TAATTTGCTGCCCTGACG	165
21	*oxc*	Oxalate-induced acid tolerance response	946845	CCGGTGACGATCGTTATTT	CTGTCGTGGTGACGTTATAG	163
22	*recA*	SOS response regulation	947170	GGTACAGCTACAAAGGTGAG	GCCTTCGCTATCATCTACAG	164
23	*recN*	Recombination and DNA repair	947105	CGATCCCAACCGACTATTTG	GTGCCTGCTGATGATGTT	194
24	*srlB*	Sorbitol permeation	948971	CCAGATGCTCATCACCTTTC	GTTCGCGAAGGTTGTCTT	176
25	*srlD*	Sorbitol-6-phosphate dehydrogenase	948937	CCTTTATCAGCGACTTCCAG	CTGTAGCCAGAGTTGTGTTT	181
26	*srlE*	Sorbitol permeation	948933	CGTGACTATGACACCAGTAAG	CCGAGACGAATGCCATAAA	163
27	*srlR*	Sorbitol repression	948942	CTGCTCTCGCGCTTTAAT	GTCGGTGCCCATAAACAA	198
28	*sulA*	Stress-Induced mutagenesis response promotion	947335	TGGGCTACCCTTAACGAA	GCTTACCGGACGCATAATAA	205
29	*tdcD*	Propionate and acetate kinase	947635	CAGCGTACGGGTTCATTT	CACCACTTTACCGTCGATAC	159
30	*tnaA*	l-Tryptophan cleavage	948221	CGATATTCCGGTGGTAATGG	GCACCATCGCATCTTTCT	164
31	*treC*	Trehalose-6-phosphate hydrolase	948762	CCCTGTATTGTGGTGCTATC	GTTGTGGTGAGGCTTCTT	152
32	*ybiJ*	Putative motility and biofilm formation factor	945433	CCAGGCACAGAACATGAATA	CCGCAGTACCGCTTAATTT	155
33	*ydeO*	Acid shock resistance	945922	GCGTAGAAGGTTCAGTCAAT	CATCGTGTTGCCCGTATT	211
34	*yebF*	Outer membrane porin formation	946363	CGTTGGGCAGATGATCAAA	GCTGATATTCCGCCATTCC	178
35	*yegR*	Hypothetical function	946613	TCAGCGTAAACTCGATAACC	TCCTACACCTTCTGTCTTCA	163
36	*yeiH*	Inner membrane formation	946668	AAGCGGAAGCCAGTAAAG	CACTTCGTGCACAGTAGAA	155
37	*yfdW*/*frc*	Oxalate-induced acid tolerance	946842	CGGAAGGCAAAGAGGTAATG	AGGCGAACACTCATCAAAC	167
38	*yfdx*	Acid shock resistance	949108	ACGATGGTCACAGCAATTC	CGTCACGCATCGCATATAA	157
39	*yhaK*	Chlorine binding and oxidative stress sensing	947620	CATATCGTGCTCGACAAAGG	GGCAACCAGCGTAATGTT	174
40	*yjiY*	Putative peptide transporter	948914	ATGAAGCGCACCCAATAC	GGTCAGTACCGTTAGCAATC	166
41	*ypdI*	Colonic acid synthesis	949107	TTTCCATTTCGGGTGGTG	GGCATTTCTTCTGTGAGGT	157

^a^ The *ffs* gene encoding a 4.5S RNA component of the signal recognition particle (SRP) is missing from this list as its size was a mere 114 bp, which was difficult to incorporate in the current scheme of RT-qPCR methodology. The gene named *mdoG* (mentioned at serial no. 18 in this table) was used as the reference gene for data analysis purposes using the Livak method (46). ID, identifier; PTS, phosphotransferase system.

### Functional importance of the genes showing altered expression in response to exogenous GSH and/or ciprofloxacin.

**FIG 4:**
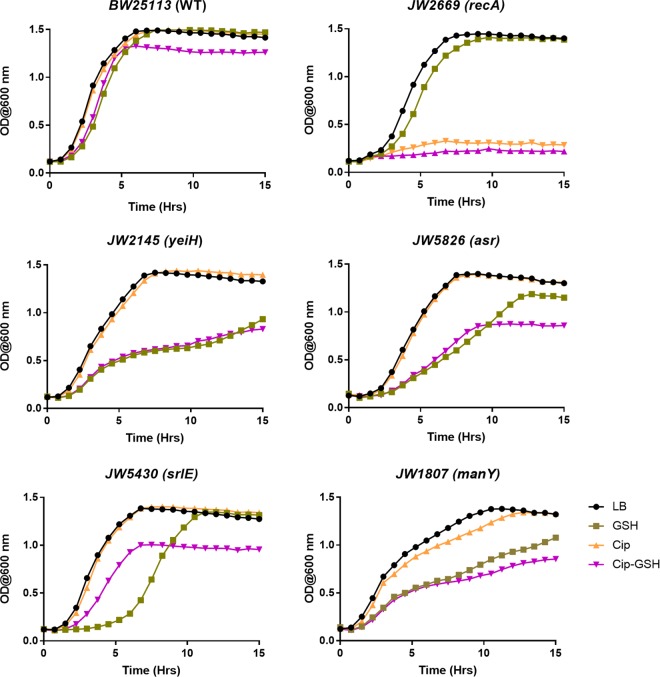
Growth kinetics of *recA*, *yeiH*, *asr*, *srlE*, and *manY* deletion strains compared to wild-type parent *E. coli* BW25113. Shown are the growth curves generated for strains BW25113, JW2669, JW2145, JW5826, JW5430, and JW1807 in LB (control); LB supplemented with 10 mM GSH (GSH); LB with 3 ng of ciprofloxacin/ml (Cip); and LB supplemented with 10 mM GSH and 50 ng of ciprofloxacin/ml (GSH+Cip).

To assess the functional importance of the genes which exhibited ≥5.0-fold-altered expression on exposure to exogenous glutathione and/or ciprofloxacin, 36 different single-gene-deletion *E. coli* mutants were picked from the Keio library (11) and their growth profiles were analyzed and compared with those of the wild-type BW25113 parent strain under GSH and ciprofloxacin exposure conditions similar to those that were used for the microarray and RT-qPCR experiments. The gene deletion mutants corresponding to the 5 remaining genes, namely, *csrB*, *fba*, *ffs*, *gcvP*, and *lexA*, were not available in the Keio gene deletion library. Of the 36 mutant strains tested in the present study, 12 strains carrying a deletion of a gene (including the *asr*, *fecA*, *manX*, *manY*, *ogrK*, *recA*, *recN*, *srlB*, *srlD*, *srlE*, *yieH*, and *yjiY* genes) exhibited a changed growth profile(s) in the presence of GSH and/or ciprofloxacin compared to wild-type parent strain BW25113. The growth curve data for BW25113 along with 5 of the mutants, namely, the *asr*, *manY*, *recA*, *srlE*, and *yeiH* deletion mutants (strains with notable deviations in their growth profiles), are shown in Fig. 4. The growth profiles of parent strain BW25113 were not very different under the tested exposure conditions, whereas the strain carrying a *recA* deletion was unable to grow wherever ciprofloxacin was present in the medium (even 3 ng/ml ciprofloxacin prevented growth of the strain). The mutants carrying deletions in *yeiH*, *asr*, and *srlE* exhibited poor growth in the presence of GSH or GSH plus ciprofloxacin. The *manY* deletion strain showed changed growth behavior in the presence of both GSH and ciprofloxacin. Among the remaining 7 strains showing moderately altered growth profiles, the *fecA*, *manX*, *ogrK*, *srlB*, *srlD*, and *yjiY* deletion mutants exhibited lower growth with the GSH sample(s) whereas the *recN* deletion mutant showed diminished growth with the ciprofloxacin samples compared to the BW25113 control strain (see Fig. S1 at http://www.barc.gov.in/publications/mSystems00001-18/).

### Glutathione supplementation leads to increased survival of *E. coli* under acidic conditions.

**FIG 5:**
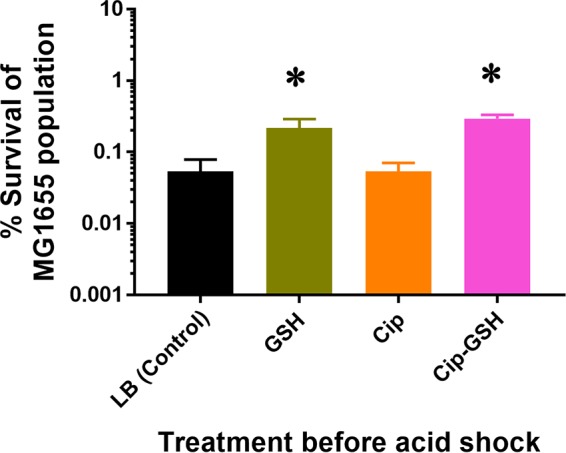
Acid resistance of MG1655 with GSH and/or ciprofloxacin at a concentration of 10 mM GSH, 3 ng of ciprofloxacin/ml (subinhibitory concentration), and 10 mM GSH with 50 ng of ciprofloxacin/ml (inhibitory concentration). Bacterial cells were grown to mid-log phase in LB broth (pH 7.0). Cells grown under the exposure conditions mentioned above were diluted 40-fold into LB broth (pH 3.0) and were incubated for 1 h at 37°C. Percent survival data represent ratios of counts of viable cells remaining after acid treatment to the viable counts before treatment. Initial cell densities (before exposure to the acidified medium) ranged from 4 × 10^7^ to 2 × 10^8^ CFU/ml. The results shown are averages from three independent experiments, with the SEMs indicated.

The exposure to GSH affected different classes of genes, including those corresponding to redox functions, transport, virulence, acid stress, and unknown functions. Though GSH is known to affect most of the biological processes mentioned above either directly or indirectly (12), acid stress adaptation vis-à-vis GSH supplementation had not been shown previously for *E. coli*. Since GSH significantly altered the expression of acid stress genes of *E. coli*, *viz.*, *asr*, *frc*, *oxc*, *ydeO*, and *yegR*, we investigated whether GSH supplementation promotes the survival of *E. coli* under acidic conditions. Survival of MG1655 at pH 3.0 was measured using the corresponding GSH and ciprofloxacin concentrations at the time of bacterial growth. In the case of the control culture, approximately ~0.05% of the initial bacterial population survived after the acid shock (Fig. 5), a level that was comparable to the survival seen with ciprofloxacin alone. On the other hand, significantly higher bacterial survival was observed in groups which were supplemented with either GSH (~0.11%) or a GSH-ciprofloxacin combination (~0.12%). The data therefore clearly signify that GSH supplementation provides a survival advantage for *E. coli* cells under conditions of an acidic environment.

## DISCUSSION

Earlier studies at our laboratory established that GSH decreases the antibacterial effect of ciprofloxacin by neutralizing antibiotic-associated oxidative stress and by promoting antibiotic efflux from *E. coli* cells (8, 9). Here we report *E. coli* genome-level expression changes in the presence of exogenous GSH *per se* and how the bacterial cells conceivably use their molecular networks to offset the effect of the ciprofloxacin by altering their stress response pathways. To the best of our knowledge, this is the first report describing the genome-wide expression changes that occur on exposure to exogenous GSH in *E. coli*. Moreover, our data offer a genomic perspective for GSH-mediated reversal of ciprofloxacin activity in *E. coli* by rationally linking the gene expression changes and functional importance of the genes with an established phenotype reported previously (8), as GSH is an important modulator of antibiotic activity in bacteria (13).

Being a prominent cellular antioxidant, GSH has a significant influence on the cellular redox state, thereby upregulating the expression of redox function genes such as *yeiH* and *azoR* in *E. coli* as observed using microarray and RT-qPCR methods. Among the genes mentioned above, *yeiH* has been previously reported to harbor an upstream *sox* box, showing its potential regulation by the *soxRS* system (14), whereas *azoR* was implicated in thiol-specific stress in a previous study (15). Genes coding for the Fe citrate transporter (*fecA*) (16) and for the Fe-S center subunit of fumerate reductase (*frdB*) (17) are downregulated by GSH, plausibly to avoid the cellular injury that occurs via Fenton reaction-induced ·OH due to elevated cellular Fe^++^ levels. Our results further indicate that GSH could be important for acid stress adaptation of *E. coli* cells. This statement is supported by our transcriptomic data showing that acid stress response genes, namely, *asr*, *frc*, *oxc*, *ydeO*, and *yegR*, are significantly upregulated on exposure to exogenous GSH. Our conclusions are supported by the fact that *asr* has been known to provide a survival advantage for bacteria under acid stress conditions (18) and the fact that the remaining four genes are critical components of acid resistance networks in bacteria (19, 20) which are regulated by *evgA* of the two-component regulatory system in *Escherichia coli*. Another *evgA*-regulated virulence gene, *emrK*, which has an established role in antibiotic efflux and biofilm formation (21, 22), was upregulated on exposure to GSH. On similar lines, GSH promoted *ypdI* expression, which is important during biofilm formation (23). GSH-mediated up- and downregulation of *yebF* and *nmpC*, respectively, could be a part of a bacterial strategy to intensify virulence by tolerating higher levels of colicins as this expression pattern has previously been implicated in such phenotypes for *E. coli* (24, 25). Similarly, GSH-mediated repression of *csrB* could contribute to augmenting the bacterial virulence (26). The results therefore suggest that high GSH levels could promote the overall virulence of *E. coli*, a finding which corroborates our previous animal model data (27) and is additionally supported by a recent independent report that GSH activates virulence gene expression in the intracellular pathogen *Listeria monocytogenes* (28). Among the virulence genes affected by GSH supplementation, *emrK* and *yebF* are also involved in transport functions in *E. coli* (22, 24). Likewise, expression of *lamB* and *srlB*, encoding transporters for different sugar derivatives (29, 30), was affected by GSH supplementation. These findings, along with the earlier reports of GSH promoting efflux of antibiotics (9, 31) and potassium (32, 33) from bacteria, imply that GSH is involved in regulating the transport function of *E. coli*. As GSH is known to be an important osmolyte for *E. coli* (12), repression of *treC* seems reasonable in the light of a previous finding (34). As per the microarray data, GSH also upregulated the expression of *tnaA*, thus increasing the intracellular levels of indole, which has an important role in antibiotic resistance development in *E. coli* (35).

Though the presence of GSH is known to be critical for a variety of biological functions, e.g., redox balance, transport, osmotolerance, etc. (12), its role in acid stress adaptation of *E. coli* is rather underexplored. In the current report, we have shown that GSH provides a survival advantage for *E. coli* under acidic conditions, though its mechanistic details are yet to be understood. Accordingly, it will be interesting to investigate whether GSH-mediated KefB/KefC activation followed by lowering of intracellular pH (32) contributes to the observed acid resistance phenomenon. Another possibility could be that of increased H_2_S formation in the medium after GSH supplementation, which might affect the pH, as H_2_S is weakly acidic in nature (36). H_2_S, being a gaseous signaling molecule, could have pleiotropic effects, including oxidative stress and antibiotic susceptibility modulation, inside bacterial cells (37, 38). The glutamic acid-activated acid resistance system (39) could also be important, as it is an integral constituent of naturally occurring tripeptide GSH.

Exposure of *E. coli* cells to ciprofloxacin at 0.1× the MIC resulted in significant upregulation of repair/recombination and cytokinesis genes, *viz.*, *dinB*, *lexA*, *recA*, *recN*, and *sulA* (the extent of the increase was shown to be ≥4.0-fold using both microarray analysis and RT-qPCR for all genes except *dinB*, which was induced by >2.0-fold). These data were in complete agreement with earlier reports describing the whole-genome changes (3) or molecule-level changes (40, 41) seen in response to exposure to fluoroquinolones for *E. coli*. The SOS response after ciprofloxacin exposure in *E. coli* mentioned above can be attributed to DNA damage produced through inhibition of DNA topoisomerase II (DNA gyrase) and topoisomerase IV activities (4, 40). Ciprofloxacin-mediated *yebF* induction in *E. coli* shows that exposure to subinhibitory levels of fluoroquinolone has a similar effect on the expression of this gene, apart from the inhibitory concentrations (3).

Analysis of transcriptomic data of *E. coli* in response to GSH plus ciprofloxacin revealed that the expression pattern of many genes is in sync with GSH exposure but with a reversal of the trend compared with that seen with the subinhibitory ciprofloxacin exposure (≥2.0-fold change cutoff value using both microarray and RT-qPCR). These abrupt changes in the level of expression offer meaningful insights about a possible mechanism(s) behind the thwarting of the action of ciprofloxacin by GSH to promote antibiotic resistance. For instance, *asr*, *yeiH*, *csrB*, *frdB*, *lamB*, and *treC* are not affected by ciprofloxacin but noteworthy upregulation of *asr* and *yeiH* and downregulation of the remaining genes was observed on exposure to GSH plus ciprofloxacin. On the other hand, the DNA damage response switched on by ciprofloxacin through induction of *recA*, *recN*, and *sulA* (40) is partially obviated by GSH, confirming its damage-shielding properties as reported previously (42). The results therefore implicate genes corresponding to redox function (*frdB*, *yeiH*), transport (*lamB*), acid shock (*asr*), DNA damage repair (*recA*, *recN*, and *sulA*), and virulence functions (*csrB*) in GSH-mediated inhibition of ciprofloxacin activity.

Significantly changed growth profiles of one-third (i.e., 12 of 36) of the single-gene-deletion mutants tested in the presence of GSH and/or ciprofloxacin prove the connection with altered expression of the same genes in wild-type parent *E. coli* under analogous exposure conditions. The results further confirm the functional importance of these genes not only at the molecular level but also at the physiological and phenotypic levels. The growth profile of most (10 of 12) of these mutants is compromised (in terms of saturation cell density and/or growth rate) under GSH exposure conditions, suggesting a vital role of these genes in GSH metabolism in *E. coli*. Though GSH is known to affect multiple pathways and networks in bacteria (12, 13, 28), a direct link of these genes with GSH adaptation or metabolism had not been reported previously for *E. coli*. The two remaining mutants (*recA* and *dinB*) are known to exhibit increased susceptibility to quinolones in bacteria (43, 44), and our data demonstrate that GSH is not able to mitigate their deficiency to restore the growth profile of *E. coli*. Genes with comparatively low levels of expression changes can sometimes also have an important bearing in terms of physiological effect as seen with the altered growth kinetics of *srlE* and *manY* mutants in the presence of GSH and/or ciprofloxacin (Fig. 4).

Taken together, the results of our present study reveal that exposure of *E. coli* to ciprofloxacin causes induction of DNA repair and cytokinesis genes. GSH, on the other hand, affects the genes related to redox function, transport, virulence, and acid stress. More than half of the GSH-influenced gene products were localized to a redox-active periplasm or membrane fraction (Table 1), implying that these are the important sites of GSH-mediated changes in *E. coli*. Our results also underscore that response to GSH and/or ciprofloxacin is complex in nature and that one stress pathway cannot be singled out, as multiple cellular responses appear to be directed toward protecting the cell against the given stressor. The type of genes affected raises the possibility that cells surviving exposure to GSH and ciprofloxacin in combination may exhibit increased resistance to other stresses and may be induced to form biofilms. We therefore conclude that GSH supplementation influences the expression of genes corresponding to multiple stress response pathways apart from its diverse physiological roles (12), including acid resistance (present study) and antibiotic activity reversal (8) via oxidative stress and efflux modulations in *E. coli* (9). Consequently, further studies are needed in this direction to understand the network of stress response pathways, including their redundancy, pleiotropy, and cross talk with antibiotic resistance triggers, for efficient management and utilization of antibiotic resources by minimizing the conditions for resistance buildup.

## MATERIALS AND METHODS

### Bacterial strains and culture conditions.

Wild-type *E. coli* K-12 strain MG1655 was used for microarray and RT-qPCR analysis. Another wild-type strain, BW25113, and derivatives of that strain carrying single gene deletions (11) were used to generate their growth profiles. A complete list of the *E. coli* strains used in the present study is given in Table 3. Frozen glycerol stocks of the bacterial cultures were streaked onto Luria-Bertani (LB) agar, and, after overnight growth at 37°C, they were stored at 4°C for short-term storage. Overnight liquid cultures (optical density at 600 nm [OD_600_], ~2.0) were inoculated into fresh LB medium (1:100), and the cells were used in mid-exponential phase (OD_600_, ~0.4 to 0.5) for microarray and RT-qPCR analysis.

**TABLE 3:** List of *E. coli* strains used

Strain name	Genotypic description	Source or reference
MG1655	F^−^ λ^−^ *rph-1*	Laboratory collection
BW25113	F^−^ Δ(*araD*-*araB*)*567* Δ*lacZ4787*(::*rrnB-3*) λ^−^ *rph-1* Δ(*rhaD*-*rhaB*)*568 hsdR514*	National Bio Resource Project (NBRP), Japan
JW1228	BW25113 Δ*adhE*::Kan^r^	Keio collection (11)
JW5826	BW25113, Δ*asr*::Kan^r^	Keio collection (11)
JW1409	BW25113, Δ*azoR*::Kan^r^	Keio collection (11)
JW0221	BW25113, Δ*dinB*::Kan^r^	Keio collection (11)
JW2365	BW25113, Δ*emrK*::Kan^r^	Keio collection (11)
JW4251	BW25113, Δ*fecA*::Kan^r^	Keio collection (11)
JW4283	BW25113, Δ*fimH*::Kan^r^	Keio collection (11)
JW4114	BW25113, Δ*frdB*::Kan^r^	Keio collection (11)
JW2237	BW25113, Δ*glpC*::Kan^r^	Keio collection (11)
JW3996	BW25113, Δ*lamB*::Kan^r^	Keio collection (11)
JW1806	BW25113, Δ*manX*::Kan^r^	Keio collection (11)
JW1807	BW25113, Δ*manY*::Kan^r^	Keio collection (11)
JW1808	BW25113, Δ*manZ*::Kan^r^	Keio collection (11)
JW5078	BW25113, Δ*nmpC*::Kan^r^	Keio collection (11)
JW2067	BW25113, Δ*ogrK*::Kan^r^	Keio collection (11)
JW2370	BW25113, Δ*oxc*::Kan^r^	Keio collection (11)
JW2669	BW25113, Δ*recA*::Kan^r^	Keio collection (11)
JW5416	BW25113, Δ*recN*::Kan^r^	Keio collection (11)
JW2673	BW25113, Δ*srlB*::Kan^r^	Keio collection (11)
JW2674	BW25113, Δ*srlD*::Kan^r^	Keio collection (11)
JW2676	BW25113, Δ*srlR*::Kan^r^	Keio collection (11)
JW5430	BW25113, Δ*srlE*::Kan^r^	Keio collection (11)
JW0941	BW25113, Δ*sulA*::Kan^r^	Keio collection (11)
JW5806	BW25113, Δ*tdcD*::Kan^r^	Keio collection (11)
JW3686	BW25113, Δ*tnaA*::Kan^r^	Keio collection (11)
JW4198	BW25113, Δ*treC*::Kan^r^	Keio collection (11)
JW0787	BW25113, Δ*ybiJ*::Kan^r^	Keio collection (11)
JW1494	BW25113, Δ*ydeO*::Kan^r^	Keio collection (11)
JW1836	BW25113, Δ*yebF*::Kan^r^	Keio collection (11)
JW5837	BW25113, Δ*yegR*::Kan^r^	Keio collection (11)
JW2145	BW25113, Δ*yeiH*::Kan^r^	Keio collection (11)
JW2371	BW25113, Δ*yfdW*::Kan^r^	Keio collection (11)
JW2372	BW25113, Δ*yfdX*::Kan^r^	Keio collection (11)
JW3077	BW25113, Δ*yhaK*::Kan^r^	Keio collection (11)
JW5791	BW25113, Δ*yjiY*::Kan^r^	Keio collection (11)
JW2373	BW25113, Δ*ypdI*::Kan^r^	Keio collection (11)

### Glutathione and ciprofloxacin solutions.

A ciprofloxacin stock solution of 2 mg/ml was appropriately diluted using sterile distilled water to adjust the final antibiotic concentration in the growth media. A fresh stock solution (250 mM) of GSH was prepared in sterile distilled water followed by ﬁlter sterilization through a 0.22-μm-pore-size membrane (Millipore) and added to the media prior to use wherever mentioned. When needed, growth medium was supplemented with ciprofloxacin and GSH at the concentrations indicated.

### Sample preparation for microarray analysis.

The microarray-determined mRNA profiles of *E. coli* MG1655 cultures in response to GSH and/or ciprofloxacin were compared with those of control or untreated cultures. Bacterial cells that had been grown overnight were freshly diluted 1:100 in LB medium for collection of total RNA. MG1655 cells were grown under three different conditions, namely, (i) with 10 mM GSH, (ii) with 3 ng/ml ciprofloxacin (subinhibitory antibiotic concentration), and (iii) with 10 mM GSH and 50 ng/ml ciprofloxacin (inhibitory antibiotic concentration). Control cultures were grown in LB with no exogenous GSH or ciprofloxacin added. All the MG1655 cultures mentioned above were collected during mid-logarithmic phase (OD_600_ of 0.4 to 0.5) for microarray analysis. Subsequently, the cells were centrifuged (8,000 × *g*, 5 min, 4°C) and suspended in RNAlater (Qiagen, USA). Two biological replicates were prepared and processed for each of the above-mentioned exposure conditions and controls.

### Microarray hybridization and analysis.

Total RNA was obtained using an RNeasy Protect Bacteria minikit (Qiagen) per the manufacturer’s instructions. The purity, concentration, and integrity of RNA were confirmed by the use of a Agilent Model 2100 Bioanalyzer and agarose gel electrophoresis. The RNA integrity number for all the samples was ≥7, confirming suitability for the microarray expression experiments. Total RNA was then reverse transcribed, biotin labeled, and purified using a GeneChip IVT express kit. Fragmented, biotinylated cDNA generated using a GeneChip IVT express kit was hybridized to Affymetrix gene chip *E. coli* genome 2.0 arrays for 16 h at 45°C and 60 rpm. Following hybridization, the hybridized probe arrays underwent an automated washing and staining protocol on an automated Affymetrix GeneChip fluidic station and were then scanned on a GeneChip scanner 3000 7G system, where patterns of hybridization were detected. All the steps of microarray hybridization and data preprocessing were performed by an Affymetrix authorized service provider, iLife Discoveries, Gurgaon, India. Raw data sets were extracted from all CEL files (raw intensity files) after scanning of slides. All the original microarray data (CEL files) for control and treated groups were preprocessed using the RMA (Robust Multichip Average) algorithm, which consists of three steps: background adjustment, quantile normalization, and summarization. All of the procedures described above were performed by selecting the RMA algorithm in GeneSpring Gx12.0. Subsequently, genes of low-intensity information content (>20 percentile) in each data set were filtered out and excluded from the downstream analysis. After this, averages of intensity values were taken for experiment sets for control and treated groups. After the data normalization, including data preprocessing, quality control (principal-component analysis), and baseline transformation steps, differentially expressed genes (DEG) were identified using the criterion that the mean difference in transcript levels was ≥2-fold with a *P* value of <0.05. Fold change was calculated by comparisons between a given condition (condition 1) and another condition or several other conditions treated as an aggregate (condition 2). Finally, the data set of significantly changed genes was subjected to hierarchical clustering for further functional enrichment using gene ontology (GO) analysis.

### Validation of gene expression using reverse transcription-quantitative PCR (RT-qPCR).

Selected gene expression results were confirmed using RT-qPCR analysis. *E. coli* strain MG1655 was grown in LB broth in the presence and absence of GSH and ciprofloxacin concentrations similar to those that were used for microarray expression analysis. Mid-log-phase cells of MG1655 (OD_600_, ~0.4 to 0.5) were used for RNA extraction. The RNA was extracted using a NucleoSpin RNA II kit (Macherey-Nagel, Germany) per the manufacturer’s instructions. The RNA was further treated with Heat and Run DNase (ArcticZymes, Norway) to remove DNA contamination and quantified using a NanoDrop 2000 spectrophotometer (Thermo Fisher Scientific, Inc., Wilmington, DE). cDNA was synthesized using a qScript cDNA synthesis kit (Quanta Biosciences, USA) and 1.0 μg of RNA in a 20-μl reaction mixture. The qPCR was performed on 3 μl of diluted cDNA (15 ng) using a Kapa SYBR Fast qPCR kit (Kapa Biosystems, USA) and a 0.1 pM concentration of each primer in a 20-μl reaction mixture. The qPCR reactions were prepared using an epMotion 5070 automated PCR Robot (Eppendorf, Germany) in 96-well format. The qPCR run and analysis were performed using a LightCycler 480 system (Roche, USA). The thermocycling conditions included one cycle of denaturation at 95°C for 4 min, followed by 45 cycles of denaturing at 95°C for 2 s and annealing/extension at 60°C for 40 s. The threshold cycle (*C*_*T*_) values were normalized against the previously reported reference gene *mdoG* encoding glucan biosynthesis protein (45), and the relative gene expression levels were determined using the ΔΔ*C*_*T*_ method (46). The 41 genes used in the study are listed in Table 2 together with their respective functions and the primers used.

### Growth profiles of Keio collection single-gene-deletion mutants.

Growth profiles of GSH-exposed and/or ciprofloxacin-exposed BW25113 and mutant *E. coli* strains lacking genes of interest obtained from the National Institute of Genetics, Mishima, Japan (11), were generated using a spectrophotometer-based approach. Overnight cultures of different *E. coli* strains were reinoculated (1:100) into a total starting volume of 10 ml LB medium, and 100 µl of these freshly diluted cultures was grown in a honeycomb 100-well plate (Bioscreen; OY Growth Curves, Finland) in a Bioscreen C machine with continuous shaking at 37°C. Absorbance readings were taken every 30 min using a wideband filter. Uninoculated LB broth was used as the control.

### Acid resistance assay.

Acid resistance was determined by the method of Masuda and Church (19), where MG1655 cells were grown to their mid-exponential phase (OD_600_ ~0.3) in the presence and absence of GSH and/or ciprofloxacin, and 50 μl of this cell culture was transferred to 1.95 ml of prewarmed LB medium (adjusted to pH 3.0 with HCl) and incubated at 37°C for 1 h. Viable cells in the acidified culture were counted by plating serial dilutions onto LB agar plates and incubated at 37°C for 16 to 18 h before colony counting was performed. The percentage of survival was the ratio of the counts of viable cells remaining after acid treatment to the viable counts before treatment.

### Statistical analysis.

The statistical analysis was performed using GraphPad Prism 7 software (GraphPad Inc., USA) either by one-way analysis of variance (ANOVA) followed by Dunnet’s posttest for multiple-group comparisons or a one-tailed unpaired Student’s *t* test for two-group comparisons. Asterisks (*) refer to a *P* value of <0.05 in comparison to control results. Though the experiments were repeated at least thrice to confirm the reproducibility of the results, the data from a single experiment were used for purposes of representation and analysis of the growth curve data. The number of replicates (*n*) for a given sample is indicated in individual figure legends.
